# Optimisation of cutaneous microbiota sampling methodology

**DOI:** 10.3389/frmbi.2025.1559981

**Published:** 2025-04-22

**Authors:** Dario Leonardo Balacco, Ajoy Bardhan, Hadeer Ibrahim, Sarah A. Kuehne, Melissa M. Grant, Josefine Hirschfeld, Adrian H. M. Heagerty, Iain L. Chapple

**Affiliations:** ^1^ Periodontal Research Group, Dentistry, School of Health Sciences, University of Birmingham and Birmingham Dental Hospital (Birmingham Community Healthcare Trust), Birmingham, United Kingdom; ^2^ School of Health Sciences, University of Birmingham, Birmingham, United Kingdom; ^3^ Epidermolysis Bullosa Unit, Department of Dermatology, University Hospitals Birmingham, National Health Service Foundation Trust, Birmingham, United Kingdom; ^4^ Faculty of Medicine, Suez Canal University, Ismailia, Egypt; ^5^ School of Science and Technology, Nottingham Trent University, Nottingham, United Kingdom; ^6^ National Institute for Health and Care Research (NIHR) Birmingham Biomedical Research Centre, University of Birmingham, Birmingham, United Kingdom; ^7^ Department of Periodontology, Operative and Preventive Dentistry, University of Bonn, Bonn, Germany

**Keywords:** skin microbiome, microbiome, swab, skin sampling, 16S

## Abstract

**Introduction:**

The cutaneous microbiome plays an essential role in guarding against invasive pathogens and maintaining healthy skin homeostasis. Several studies have demonstrated the importance of a healthy skin microbiome through its alteration in several diseases. Differing skin characteristics across the body (temperature, pH, humidity) create distinct ecological niches inhabited by diverse microbial communities. The study of cutaneous microbiota is further complicated by numerous variables at all stages of investigation, including study design, skin sampling method, sample storage, sample processing, sequencing, and data analysis. Utilisation of standardised approaches is critical for reproducibility and comparison between skin microbiome studies. However, there is a notable lack of standardisation of sampling methodologies in the literature. Studies have employed differing sampling strategies and conditions which may affect microbiota characterisation.

**Methods:**

Antecubital fossa was sampled from sixteen individuals using sterile dry cotton swabs or eSwabs. Sterile phosphate buffered saline, or 0.9% sterile saline were used as moistening solutions. Samples were then either stored at room temperature for 30 minutes or stored at -80°C for at least 24 hours before processing. Cutaneous microbiome was identified using 16S sequencing.

**Results:**

Comparative analysis determined whether the type of swab (cotton/eSwab), moistening solution (saline solution/phosphate buffered saline), duration of swabbing (30 sec/1 min), and sample storage temperature (room temperature/-80°C) affect sampling and identification of skin microbial communities. Comparison of the total DNA yield extracted using different conditions showed that while moistening solution, duration of swabbing, and storage conditions did not affect the total DNA amount, using eSwabs yielded higher biomass.

**Discussion:**

Sampling approaches are critical for the success of sequencing. The conditions investigated in this study did not influence microbiome profiling allowing consistent sampling of the microbiota. However, data clustering was affected more by individual subject than by the conditions investigated, suggesting the importance of recognizing inter-individual variability as an important factor in real-life skin microbiome studies.

## 1 Introduction

Over the last 25 years, the study of skin microbes and their relationships with human hosts has attracted growing interest, aided by advances in sequencing technologies and bioinformatic tools. The scientific community has investigated the cutaneous microbiome in health and disease, including atopic dermatitis, acne vulgaris, psoriasis, hidradenitis suppurativa, seborrheic dermatitis, rosacea, lichen sclerosis, and alopecia areata (Langan et al., 2020; Chattopadhyay et al., 2021; Carmona-Cruz et al., 2022; Condrò et al., 2022; Sánchez-Pellicer et al., 2022). Besides the technological advances, uniform study design, standardised methods, methodological validation, and reproducible data analysis are important features for accurate interpretation and comparison of microbiome studies (Kong et al., 2017). The rigour necessary for reproducible sampling and analysis can render skin microbiome research an arduous undertaking. Indeed, the assiduousness required has prompted this dynamic to be referred to as ‘a method to the madness’ (Kong et al., 2017).

Studies are further complicated by the diversity of the ecological niches that microbes inhabit within specific sites of the human body; the cutaneous microbial communities not only vary between individuals but also between body sites within the same individual according to local skin properties (eg. oily, moist, dry, pressure bearing area), as reviewed in Byrd et al., 2018. Moreover, metagenomic analyses of human skin microbiomes are associated with very low microbial biomass (generally in the pg and ng range), high risk of contamination, and complexity in isolating enough DNA for sequencing and downstream analyses (Kong et al., 2017; Byrd et al., 2018; Bjerre et al., 2019; Bay et al., 2020). Optimised methodologies have been developed; nevertheless, they are affected by numerous variables in all stages, including study design, but also skin sampling method, sample storage, sample processing, sequencing method, and data analysis (Kong et al., 2017).

Employing standardised approaches is critical for ensuring reliable, reproducible, and comparable skin microbiome studies. Sampling techniques such as skin biopsies, tape stripping, skin scrapes, and skin swabs differ in biomass yield, human DNA contamination, discomfort, and ultimately microbial communities identified (Kong et al., 2017). Commercially available kits for skin microbiome DNA extraction have been developed with different strategies to disrupt cells, such as thermal lysis and enzymatic or mechanical disruption. Bjerre et al., 2019 compared 12 commercially available kits highlighting different characteristics, success rates, and isolation of microbial communities. The study concluded that choice of DNA extraction strategy affects the microbial profile, but no more than inter-individual variation.

Despite a “manual of procedures” being published in 2012 (Methé et al., 2012), we found marked heterogeneity in the scientific literature on the type of swabs employed for microbiota sampling, such as catch-all swabs (Aagaard et al., 2013; Chng et al., 2016; Naik et al., 2020; Moitinho-Silva et al., 2022), eSwabs (Riverain-Gillet et al., 2020), or cotton swabs (Schowalter et al., 2010; Ogai et al., 2018; Schneider et al., 2022; Zhou et al., 2022). In addition, we found variability in the moistening solutions employed and in the duration of swabbing (ranging from 20 seconds to 1 minute and from 10 times to 100 times) (**Table 1**).

**Table 1:** Sampling methods details reported in literature.

Article	Type of swab	Moistening solution	Duration	Storage (°C)
Schneider et al., 2022	–	–	–	–
Hsieh et al., 2022	–	–	–	–
Li et al., 2019b	–	–	10 times	-20
Ahluwalia et al., 2019	–	0.15 M NaCl with 0.1% Tween 20	–	-20
Capone et al., 2022	–	0.9% NaCl	30 sec	-80
Koike et al., 2020	BD BBD culture swab plus	–	>=50 times	-80
Klymiuk et al., 2016	BD Culture SwabsTM EZ	0.15 M NaCl with 0.1% Tween 20	–	-80
Dimitriu et al., 2019	Catch-all	50 mM Tris, 1 mM EDTA, 0.5% Tween 20	30 sec	-80
Naik et al., 2020	Catch-all	Lysis buffer	20 sec	-80
Oh et al., 2014	Catch-all	MasterPure™ Yeast DNA Purification Kit Lysis buffer	–	-80
Findley et al., 2013	Catch-all	MasterPure™ Yeast DNA Purification Kit Lysis buffer	–	–
Chng et al., 2016	Catch-all	PBS+0.1% vol/vol Triton-X100	60 sec	–
Selway et al., 2020	Catch-All	0.9% NaCl	30 sec	-20
Moitinho-Silva et al., 2022	Catch-all	Specimen collection fluid	–	-80
Aagaard et al., 2013	Catch-all	Tris-EDTA and 0.5% Tween 20	30 sec	-80
Riverain-Gillet et al., 2020	COPAN 167C swabs	Ultrapure DNase-free distilled water	60 sec	-80
Susic et al., 2020	Copan eNat	Guanidine thiocyanate-based DNA stabilising medium	–	-80
Liu et al., 2020	Copan flock	DNA-free water	100 times	–
Salava et al., 2017	COPAN FLOked	0.15 M NaCl with 0.1% Tween 20	20 times	-80
Verbanic et al., 2022	COPAN FLOQ	PBS	30 sec	4
Liang et al., 2022	COPAN FLOQ	0.9% NaCl	3 times	–
Wetzels et al., 2021	cotton	–	–	-20
Woo et al., 2020	cotton	–	–	–
Kang et al., 2021	cotton	–	–	–
Kelhälä et al., 2018	cotton	–	10 times	-20
Maruyama et al., 2022	cotton	–	3 times	–
Li et al., 2019a	cotton	–	60 sec	-20
Jugé et al., 2018	cotton	0.15 M NaCl with 0.1% Tween 20	–	-20
Redel et al., 2013	cotton	0.15 M NaCl with 0.1% Tween 20	30 times	-80
Mougeot et al., 2022	cotton	0.15 M NaCl with 0.1% Tween 20	45 sec	-80
Staudinger et al., 2011	cotton	0.15 M NaCl with 0.1% Tween 20	60 sec	–
Tian et al., 2022	cotton	0.15 M NaCl with 0.1% Tween-20	30 sec	-80
Zhou et al., 2022	cotton	0.9% NaCl	30 sec	-80
Khayyira et al., 2020	cotton	0.9% NaCl and 0.1% Tween-20	–	–
Ogai et al., 2018	cotton	0.9%NaCl+ 0.1% Tween-20	–	-80
Schowalter et al., 2010	cotton	dry	–	–
Ahle et al., 2020; Ahle et al., 2021	cotton	Na_2_HPO_4_ (12.49 g/L), KH_2_PO_4_(0.63 g/L), 0.1% Triton X-100	–	-20
Potbhare et al., 2022	cotton	PBS	–	-80
Peng and Biswas, 2020	cotton	PBS	–	–
Huang et al., 2022	cotton	0.9% NaCl	–	-80
Han et al., 2018, 2018	cotton	0.9% NaCl	50 times	-80
Akaza et al., 2022	cotton	TE	–	-20
Hall et al., 2018	cotton	Yeast lysis buffer	–	-80
Oates et al., 2012	Dual Amies transport swabs	0.9% NaCl	–	–
Burnham et al., 2016	eSwabs	–	–	–
Ring et al., 2019	eSwabs	–	–	-80
Hwang et al., 2021	eSwabs	50 mM Tris, 1 mM EDTA, 0.5% Tween 20	30 sec	-80
Assarsson et al., 2018	eSwabs	Liquid Amies solution	–	–
Bjerre et al., 2019	eSwabs	Preservation medium or enzymatic lysis buffer.	30 sec	-80
Mills et al., 2022	FLOQ swab	0.9% NaCl	30 sec	-20
Proctor et al., 2021	foam swabs	Yeast Cell Lysis solution (Lucigen)	–	-80
Bayal et al., 2019	HiCulture Sterile	0.15 M NaCl with 0.1% Tween 20	50 times	-80
Kurosaki et al., 2020	HydraFlock	0.9% NaCl	-	4
Loesche et al., 2018	Polyester-tipped	0.9% NaCl	–	–
Yu et al., 2018	Rayon swabs	–	30 sec	4
Dekio et al., 2005; Dekio et al., 2007	Sterile (Nissui)	Dilution liquid	30 sec	–
Schneider et al., 2022	synthetic fibre swab	Yeast lysis buffer	-	-80

The table summarises details of methods use to swab skin for microbiome studies reported in a selection of scientific studies. Specifically, type of swab, moistening solution, duration of swabbing, and storage condition are reported. Literature review was performed using the terms “SKIN” “MICROBIOME” “SWAB” on Pubmed and returned 122 results. “-” denotes no information was provided.

Identifying optimal conditions prior to sampling and subsequent DNA extraction is challenging, time-consuming, and critical for successful microbiome metagenomic analysis. In this study we performed a comparative analysis to determine whether a subset of conditions not previously systematically reported in the scientific literature impact upon skin microbiome analysis.

To our knowledge, this is the first study to compare the effects of type of swabs, moistening conditions, duration of swabbing, and sample storage temperature upon the collection and identification of skin microbial communities using 16S sequencing. We determined that the reported skin swabbing strategies do not affect microbiota sampling under the conditions investigated. This is an important finding that suggests generalisability of findings from the existing cutaneous microbiome literature.

## 2 Results

### 2.1 Type of swab affects total biomass yield

**Figure 1:**
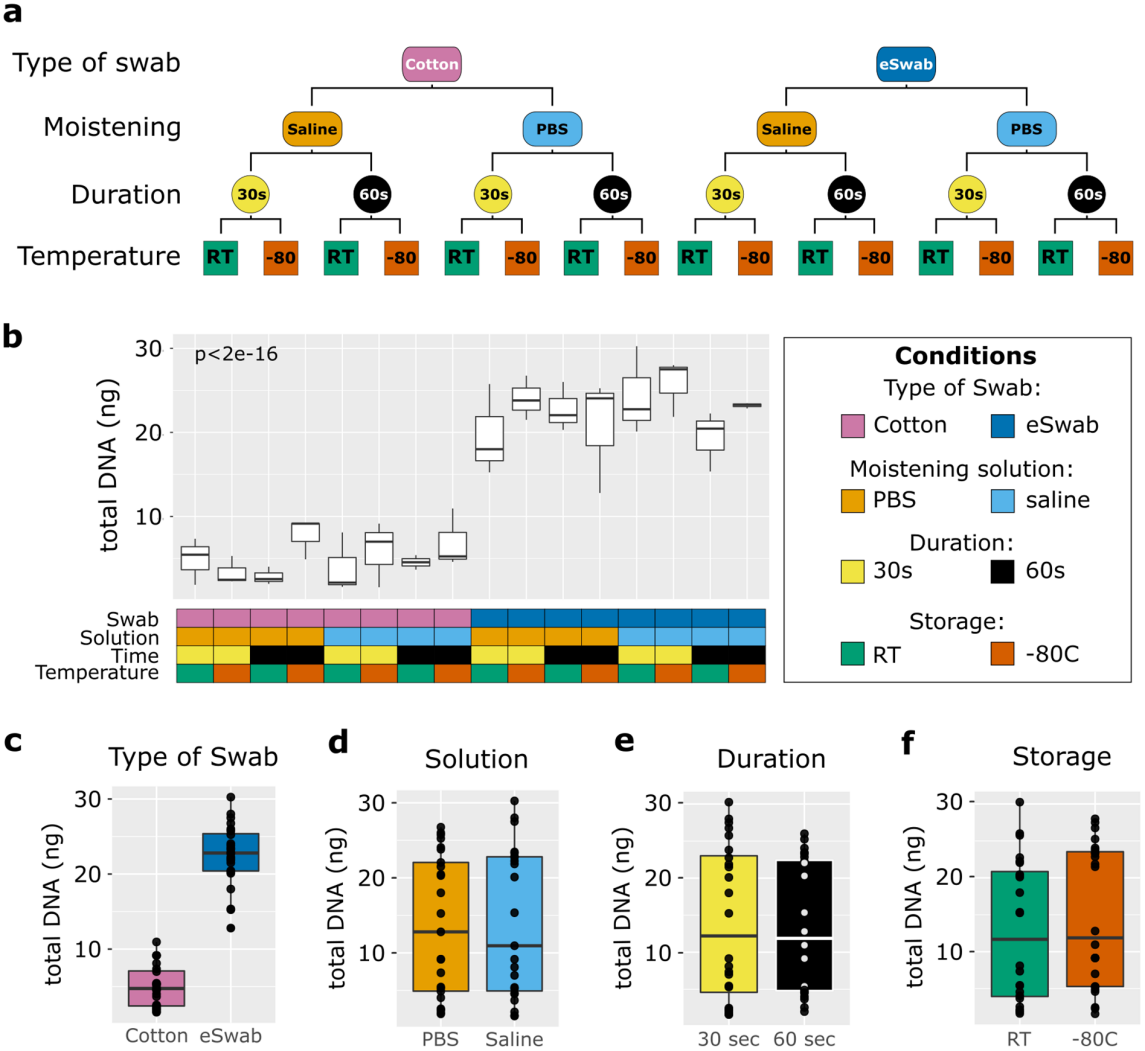
Study design and total biomass extracted. **(a)** The scheme shows the study design. Four conditions were investigated: type of swab (cotton vs eSwab), moistening solution (0.9% saline vs Phosphate buffered saline-PBS), duration of swabbing (30 seconds vs 1 minute), and storage condition (Room temperature vs -80°C). **(b)** Total biomass yield expressed as total DNA (ng) extracted was measured for each of the conditions investigated, which are summarised in the grid below and colour-coded. The effects on the total DNA yield were also investigated by condition separately such as **(c)** type of swab, **(d)** moistening solution used, **(e)** duration of swabbing, **(f)** storage conditions.

Sixteen healthy volunteers (12 females, 4 males) aged 18 to 46 years were included in the study. In total, 48 samples were collected. We utilised different conditions to sample microbial communities from the antecubital fossa: cotton swabs/flocked nylon swabs (eSwabs); pre-moistened with saline solution/PBS; swabbing for 30 seconds/1 minute; and storage at RT/-80°C (**Figure 1a**). Total DNA was extracted from the swabs and quantified using a Qubit, as described in the methods section. The DNA yield extracted from the swabs ranged between 1.87 ng and 30.25 ng, in agreement with (Bjerre et al., 2019, whose study isolated DNA between 0.3 and 29.7 ng. Significant differences in concentrations of isolated total DNA were observed using different conditions (**Figure 1b**). Cotton swabs yielded an average of 5 ng in total (range 1.87 to 10.95 ng). In contrast, eSwabs yielded an average of 22.48 ng (range 12.8 to 30.25 ng) (**Figure 1c**). The moistening solution employed (**Figure 1d**), duration of swabbing (**Figure 1e**), and storage conditions (**Figure 1f**), did not affect average total DNA yield.

Microbial communities were identified with 16S rRNA gene sequencing (BioProject PRJNA940670). Library preparation failed for four samples, not correlated with the conditions used. In fact, among these four, one sample was collected with cotton swabs using PBS for 1 minute and directly processed at RT, one sample was collected using eSwab and PBS for 30 seconds and processed at RT, one sample was collected using eSwab and saline solution for 30 seconds and stored at -80°C, and one sample was collected with eSwab using saline solution for one minute and processed at RT (**Supplementary Table 1**). In total 11,488,003 reads were generated for the 44 samples. The minimal number of reads in a sample was 37,506, the maximum 457,005, and with a median of 270,318.

### 2.2 Skin swab strategies captured similar microbial communities

**Figure 2:**
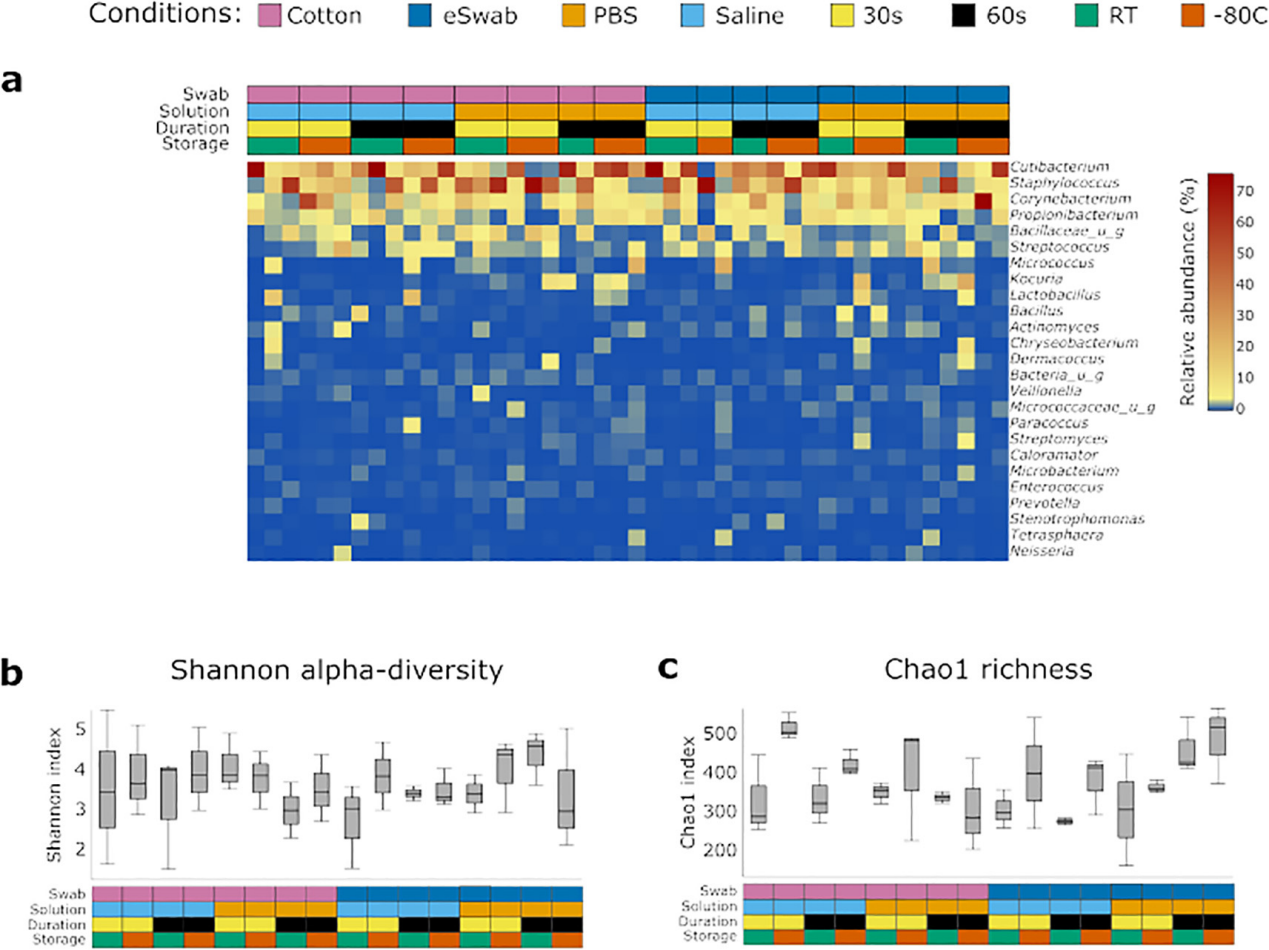
Identification of bacterial communities. **(a)** Bacterial profiles of each sample were obtained by 16S sequencing. The heatmap shows the relative abundance of the most 25 abundant genera. The conditions used to collect the samples are summarised in the grid above and are colour-coded. Diversity indexes were calculated for the microbial profile collected using the investigated conditions: **(b)** Shannon alpha-diversity index, **(c)** Chao1 richness index. The conditions used to collect the samples are summarised in the grid below and are colour coded.

**Figure 3:**
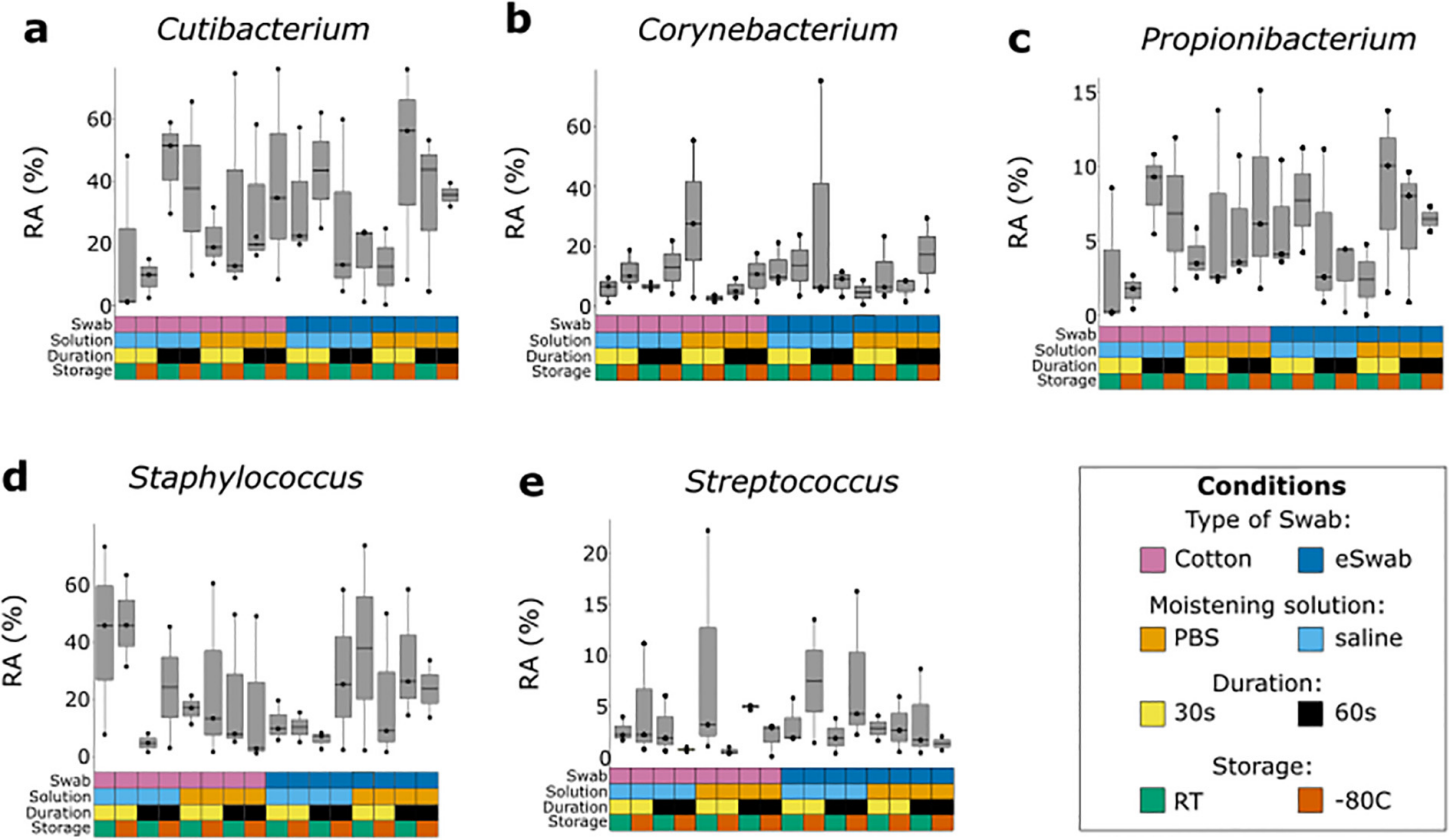
Relative abundance of most common bacterial genera sampled with different conditions. Each genus was investigated using different sampling conditions. Each box and whisker plot represents the relative abundance (RA) of a specific genus sampled from three different individuals using a specific condition, indicated by the grid below and colour-coded according to the legend. **(a)** Relative abundance of *Cutibacterium*. **(b)** Relative abundance of *Corynebacterium*. **(c)** Relative abundance of *Propionibacterium*. **(d)** Relative abundance of *Staphylococcus*. **(e)** Relative abundance of *Streptococcus*.

We investigated the microbial communities from samples collected under different conditions without identifying any major differences across groups. Independently of the conditions employed to take samples, the most abundant operational taxonomic units (OTUs) were *Cutibacterium*, *Staphylococcus*, *Corynebacterium*, *Propionibacterium*, *Bacillaceae* (undefined group) and *Streptococcus* (**Figure 2a**), with a relatively minor impact on Shannon-alpha Diversity (**Figure 2b**), and Chao1 richness (**Figure 2c**) indices (**Supplementary Data TS1**). Linear regression analysis of Shannon and Simpson indices did not highlight any significant difference. Furthermore, to identify the impact of the investigated conditions on the sampling of selected genera of bacterial residents commonly found on the skin, we compared their relative abundance sampled using different conditions (**Figure 3**). We investigated the relative abundances of *Cutibacterium* (**Figure 3a**), *Corynebacterium* (**Figure 3b**), *Propionibacterium* (**Figure 3c**), *Staphylococcus* (**Figure 3d**), and *Streptococcus* (**Figure 3e**). While we could detect inter-individual variability of the selected genera, no significant difference between conditions was observed. Evaluation of beta-diversity using Bray-Curtis and Jaccard dissimilarity did not reveal any clustering by conditions (**Supplementary Figures S1**, **S2**).

### 2.3 Inter-individual variability

**Figure 4:**
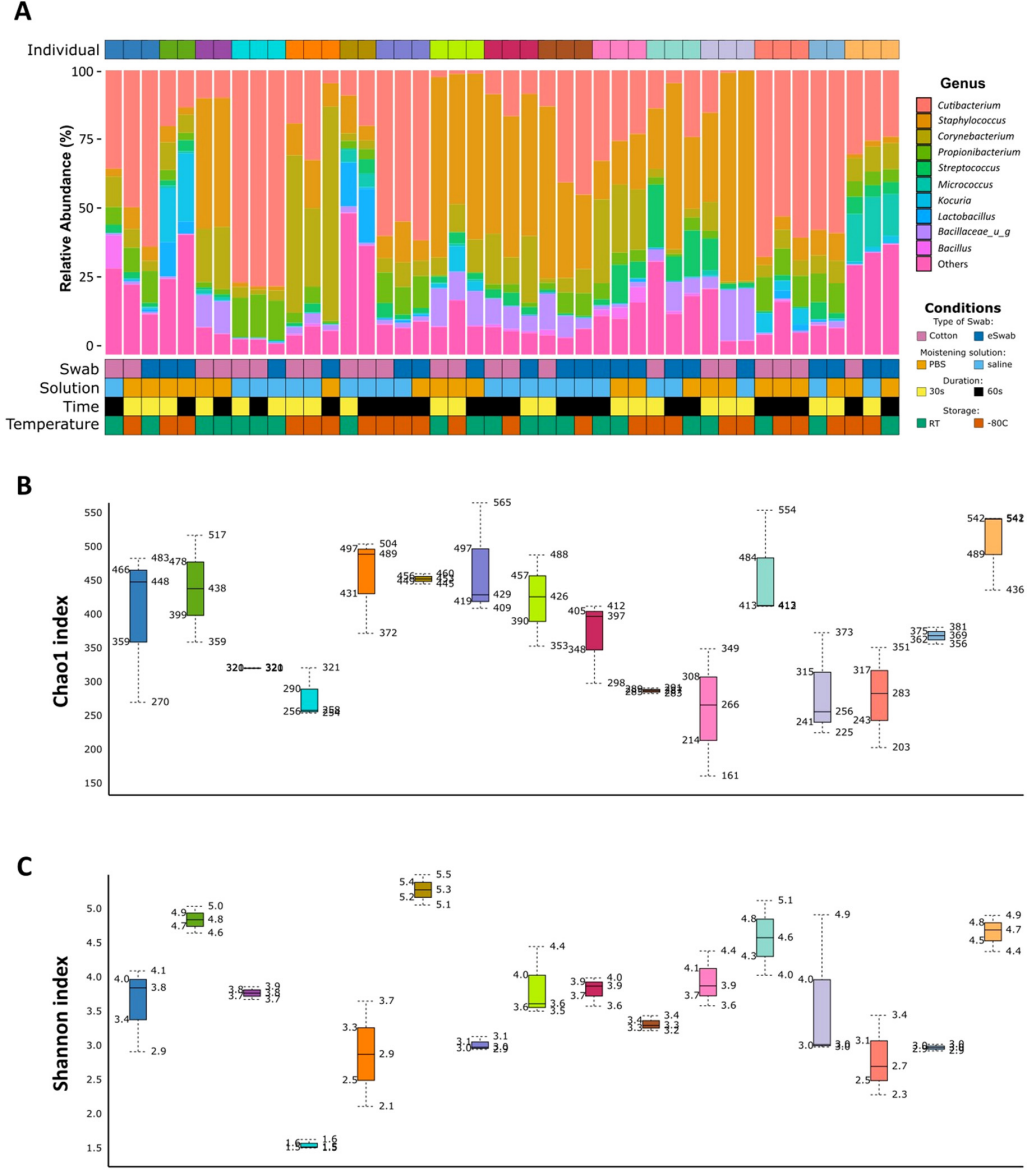
Inter-individual variability. **(a)** Relative abundance (%) of the 10 most abundant identified genera for each sample. Each individual is identified by a different colour in the top grid. Conditions used for sampling are identified for each sample and shown in the bottom grid. **(b)** Chao1 diversity index is shown for each patient. Each box represents an individual and is indicated by a different colour. **(c)** Shannon diversity index is shown for each patient. Each box represents an individual and is indicated by a different colour.

Inter-individual variability was considered in the proposed studies. We clustered the samples by similarity (**Supplementary Figure S3**) and performed PCA (**Supplementary Figure S4**), observing that samples clustered by patient ID regardless of the condition used. We investigated the relative abundance of the 10 most abundant genera for each sample and grouped by individual to visualise inter-individual variability among samples (**Figure 4a**). In addition, Shannon and Chao1 diversity indices showed consistency of the individual microbiome profile (**Figures 4b**, **c**). We observed differences between individuals not dependent upon type of swab, moistening solution, duration, nor storage. Evaluation of beta-diversity using Bray-Curtis and Jaccard dissimilarity supported grouping by patient ID (**Supplementary Figures S5**, **S6**).

## 3 Discussion

Human skin is inhabited by a complex variety of microorganisms, including bacteria, fungi, and viruses. The cutaneous microbiome plays a pivotal role in maintenance of skin homeostasis and immune responses. Variations of the skin microbiome are observed between different body sites according to their skin properties (eg. oily, moist, dry, and foot) (Byrd et al., 2018; Nathan et al., 2023). Characterisation of the cutaneous microbiome at an eubiotic state is essential to understand the changes observed in disease states. Nonetheless, different sampling and sequencing approaches can affect microbiome profiling, making the use of standardised operative procedures necessary. However, regardless of the availability of a “manual of procedures” provided by the microbiome consortium (Methé et al., 2012), studies have used heterogeneous sampling approaches.

For this reason, we compared a subset of the conditions used for sampling the cutaneous microbiome utilising swabs with subsequent 16S ribosomal RNA gene sequencing. Previously Bjerre et al., 2019 extensively studied the effect of sampling strategies and DNA extraction methods, comparing eSwabs and skin scrapes, body sites, and several DNA extraction kits. This study found that data collected using eSwabs were more consistent. In addition, Chng et al., 2016 compared tape stripping, Catch-All Sample Collection Swab, and cup scrub sampling methods, while Grice et al., 2008 compared cotton swabs, scrapes, and punch biopsies. Swabs are the least invasive technique and therefore may be the preferred method for certain indications, acceptability to participants, or disease states where risks of overt infection, skin fragility or delayed healing may be considerations. Klymiuk et al., 2016 compared the effects of storage periods (24h, 90 days, and 365 days at -80°C) of skin swab samples in 8 healthy individuals in three body sites. The study found no differences in richness nor Shannon indices among the groups. Interestingly, differences in the ratios of the most abundant taxa at different time points and different body sites were observed. However, the authors state that while these results may indicate biological differences, they could also represent technical artifacts caused by different DNA isolation kit batches used over time.

In our study, we investigated whether the type of swab used, moistening solution, duration of swabbing, and storage temperature affect microbiome sampling (**Figure 1**). Notably, other conditions such as body site and pressure of swabbing may cause different biomass yields, and further studies are required to determine their impact. However, to limit the impact of these factors in our study, we sampled a single body site (antecubital fossa), and only two researchers collected the samples.

We compared the total DNA yield extracted using different conditions and found that while moistening solution, duration of swabbing, and storage conditions did not affect the total DNA amount (**Figure 1**), using eSwabs yielded higher biomass (**Figure 1c**). Importantly, the total DNA yield included human DNA, an essential factor to consider as contamination by host DNA may interfere with the downstream analysis, especially when performing metagenomic sequencing (Pereira-Marques et al., 2019). Pereira-Marques et al. have demonstrated that high levels of host DNA decrease the sensitivity of whole genome sequencing for microbial profiling, in particular affecting the detection of low-abundant organisms. Whilst host DNA depletion methods can be effective to improve microbial profiling, they also pose disadvantages, such as the requirement of fresh samples, intact living bacteria, high molecular weight intact DNA, and introduce a bias favouring high CpG methylated bacteria (Shi et al., 2022). Computational filtering of human genome-mapped reads is commonly used to remove host contamination; however, increased computational power is required and sequencing sensitivity will be reduced. This is an important factor to consider when selecting the sampling methodology and sequencing approach, especially when investigating fragile skin diseases, where a higher percentage of host DNA compared to healthy individuals is expected. Limiting the collection of human skin using cotton swabs rather than eSwabs may benefit the overall investigation. However, further investigations, such as 16S copy number quantification, are required to determine the relative proportions of human and microbial DNA collected via sampling by cotton swabs and eSwabs respectively. We hypothesise that eSwabs, being rougher on the surface, harvest more human cells than cotton swabs, increasing the total DNA yield. However, in our study, we did not find any correlation between the yield of total DNA (including host DNA) and number of microbial reads, richness and evenness indexes.

In this study, microbial communities were investigated using 16S sequencing. We did not identify any significant differences in diversity indices using different conditions (**Figure 2**). Lauber et al., 2010 have previously investigated the effect of storage temperature and duration of storage on microbial profiling assessed using barcoded pyrosequencing of 16S rRNA without identifying any differences between these parameters. This is in keeping with our findings obtained using Illumina technologies, which are now routinely employed in sequencing studies. In addition, as the data presented in this study were collected from several patients, we performed an inter-individual analysis to investigate the variability of the samples among individuals (**Figure 4**). In fact, studies have shown inter-individual differences in the cutaneous microbiome despite matching for body site and age (Kong et al., 2017). The analysis highlighted not only variability between individuals, but also intra-individual variability. While we investigated robustness of sampling methods in a cohort with several individuals to ensure our data was generalisable to real-life studies, a study design evaluating the effects of sampling methods on the skin microbiome within the same individuals would provide further important insights. We observed heterogeneity of skin microbiome composition between individuals; therefore, our statistical analyses were performed using a mixed model for multiple comparisons to account for samples obtained from the same or different individuals. We acknowledge that our study has limitations such as the inclusion of a cotton swab, without skin contact, moistened with saline solution and frozen at -80°C as a negative control and a lack of negative controls for all the remaining conditions, no standardisation of skin preparation, the use of buffers without detergents due to the concern of the effects of detergents on some patient populations, and small sample size. Notably, library preparation of four samples failed; no clear cause was identified. In addition, our results are specific to the antecubital fossa, and the variables investigated may have a higher impact upon other body sites. It therefore remains fundamental to include a detailed description of the sampling strategy in all cutaneous microbiome investigations.

## 4 Conclusions

The conditions investigated in this pilot study can be used interchangeably to study the skin microbiome. Cotton swabs may be preferable in studies that involve fragile skin diseases as they yielded less total biomass thereby limiting host contamination without affecting downstream sequencing and bioinformatics analyses. Sampling approaches are critical for the success of sequencing, and other conditions such as DNA extraction method, study design, and bioinformatic pipeline used add substantial variation to skin microbiome studies. Data clustering was affected more by individual subject than by the conditions investigated, suggesting that, whilst the conditions investigated do not impact microbiota sampling, it is important to recognise inter-individual variability as an important factor in real-life skin microbiome studies.

## 5 Materials and methods

### 5.1 Study design

Sixteen individuals aged between 18 to 46 years with no history of inflammatory disorders (including skin disease), not taking systemic medications nor applying prescribed topical antimicrobials or topical steroids were selected via verbal screening and medical history taking. Ethical approval (Reference 19/SW/0198) and written informed consent from participants were obtained. Individuals were screened during the middle of the working day/lunchtime so that no recent showering or bathing would have taken place. Each individual was asked to donate 3 swabs with 3 different conditions randomly assigned using R, for a total of 48 samples, to ensure collection in triplicates for each condition and inclusion of interpersonal variability. Swabs were collected from the antecubital fossa from non-overlapping areas of about 1 cm^2^. Each of the three samples donated by the same individual were 1 cm apart on the skin surface. We used sterile dry cotton swabs (MWE, Cat. MW102) and eSwabs (COPAN, Cat. 480C). Sterile phosphate buffered saline, or 0.9% sterile saline were used as moistening solutions; samples were stored at room temperature for 30 minutes, then processed or stored at -80°C for at least 24 hours (**Figure 1a**). To limit pressure variability and technical differences, swabbing was performed only by authors DLB and AB, and according to a standard operating protocol.

The negative control included in the study was obtained using cotton swabs from the same batch of swabs, without skin contact, moistened with saline solution and frozen at -80°C (**Supplementary Figure S3**).

### 5.2 DNA extraction

DNA was extracted from skin swabs using the QIAmp DNA Investigator kit (Qiagen, Cat. 56504) and QIAshredders homogenisers (Qiagen, Cat. 79656). DNA was quantified using DNA HS kit (ThermoFisher, Massachusetts, USA) and Qubit (ThermoFisher, Massachusetts, USA). All kits were used according to the manufacturers’ instructions.

### 5.3 16S sequencing and taxonomic profile

Isolated DNA was shipped to CosmosID (Germantown, MD, USA) for library preparation and sequencing. Vendor optimised protocol was used. Briefly, genomic DNA was amplified via PCR with primers 27F (AGAGTTTGATCCTGGCTCAG) and 534R (ATTACCGCGGCTGCTGG) covering hypervariable regions V1 and V3. Final libraries’ quantity and quality were assessed by Qubit 2.0 (ThermoFisher, Massachusetts, USA) and TapeStation D1000 ScreenTape (Agilent Technologies Inc., California, USA) respectively. Sequencing was performed on Illumina^®^ Miseq (Illumina, California, USA) with a read length per sample of 500K in each direction.

For taxonomic profiling based on amplicon data, the CosmosID 16S data analysis was used (CosmosID Inc, 2022). Briefly, raw reads were first trimmed to remove adapters and bases of low quality and forward and reverse overlapping pairs were joined. OUTs were assigned using the CosmosID curated 16S database using a close-reference OUT picker and 97% sequence similarity through the QIIME framework. Data exploration and visualisation, and comparative analyses were performed using the CosmosID-Hub(CosmosID Inc [no date]).

### 5.4 Statistical analyses

Linear regression using mixed model multiple comparisons was performed in R using the lm4, lmerTest and multcom packages using Tukey’s Honest Significant Difference (HDS) test. Microbiome statistical analyses were performed by using the CosmosID comparative analyses tool (CosmosID Inc). Alpha-diversity and richness were calculated using Shannon index, Simpson index, and Chao1 richness. Beta diversity was estimated as Jaccard and Bray-Curtis divergence and compared by permutational multivariate analysis of variance (PERMANOVA). For all analyses, *p* ≤ 0.05 was considered statistically significant.

## Data Availability

The original contributions presented in the study are publicly available. This data can be found here: NCBI BioProject, accession PRJNA940670.
