# Supplementary figures and images for: Optimisation of cutaneous microbiota sampling methodology
Source: Front Microbiomes. 2025 Apr 22;4:1559981. doi: 10.3389/frmbi.2025.1559981 (PMC12993629; doi:10.3389/frmbi.2025.1559981)

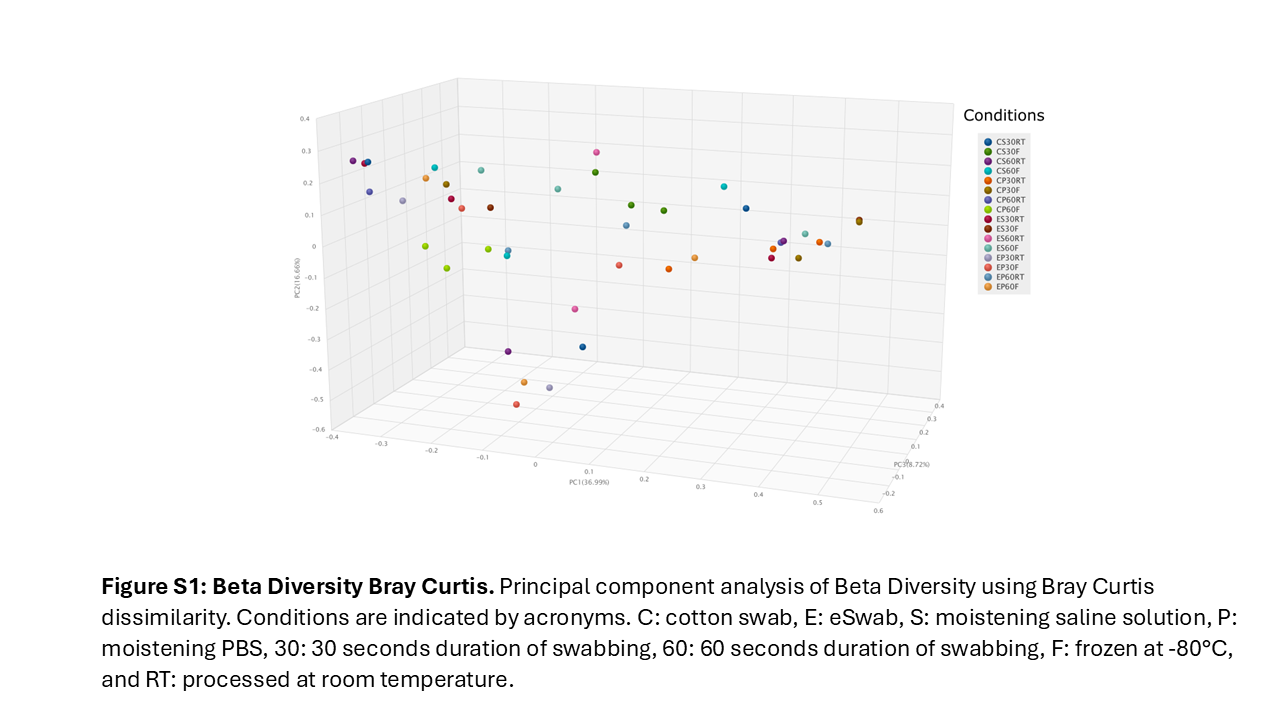

Supplement: Supplementary file 1 [file Image1.tif]

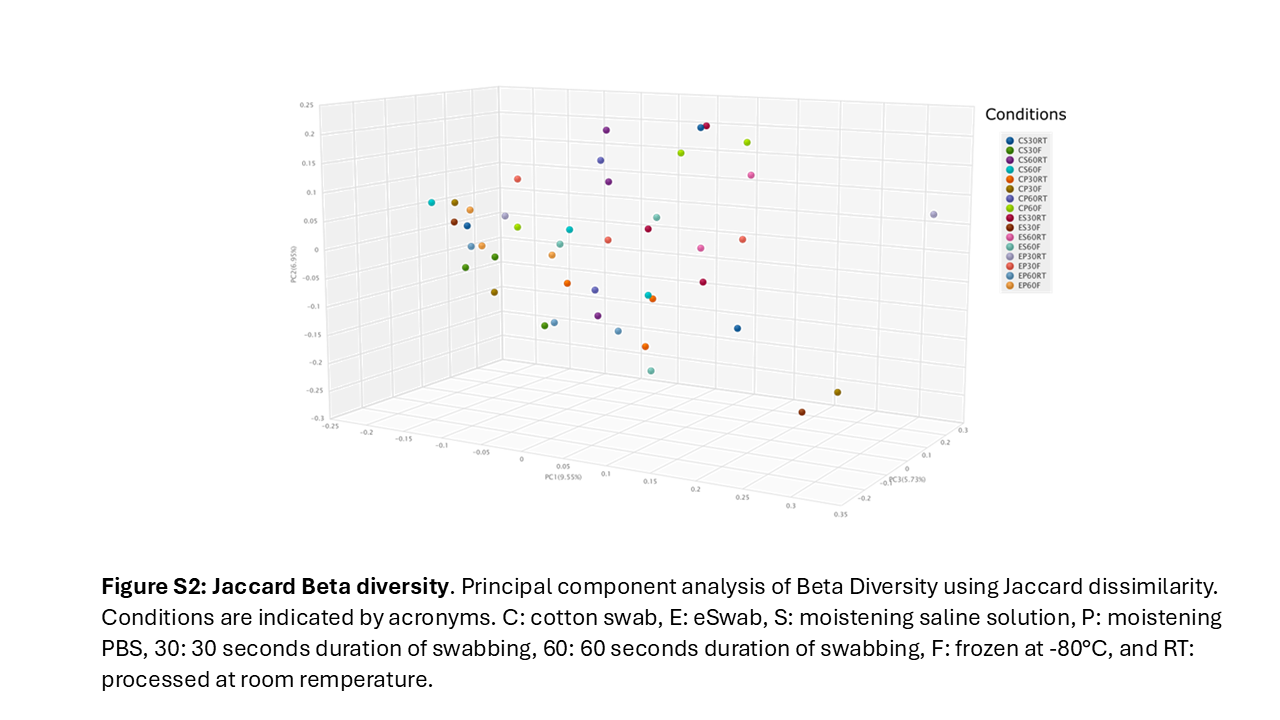

Supplement: Supplementary file 2 [file Image2.tif]

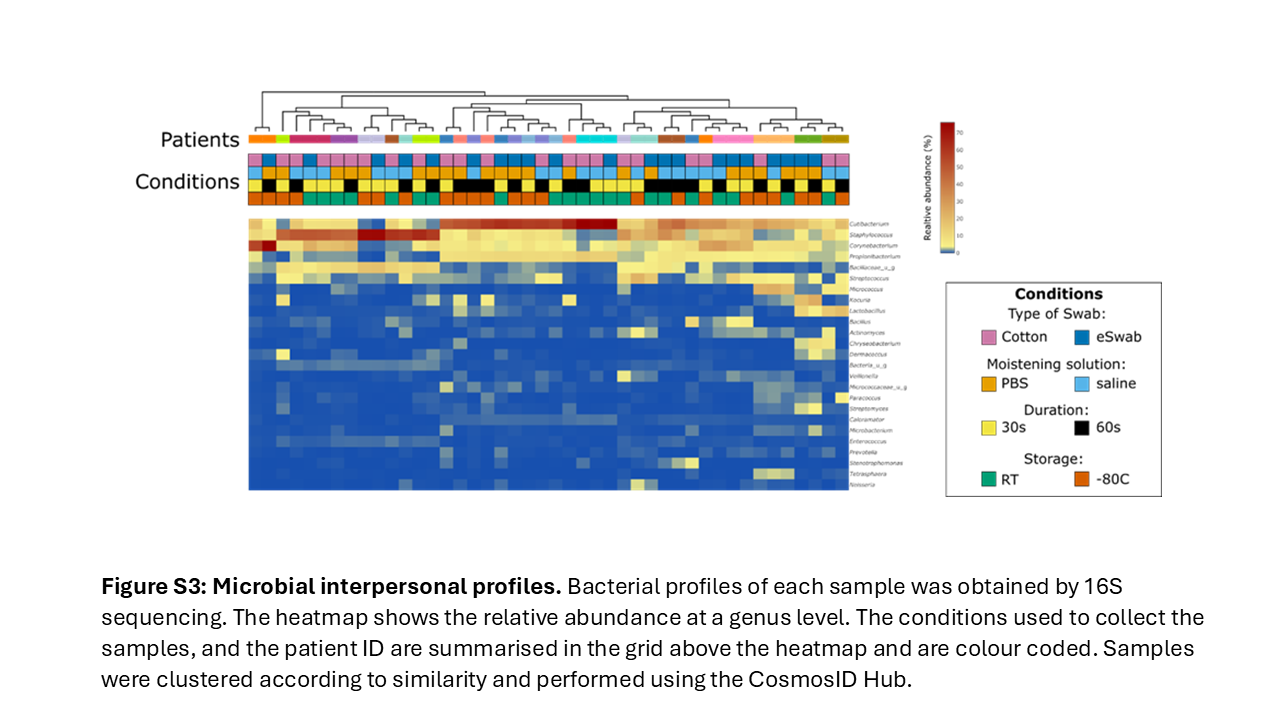

Supplement: Supplementary file 3 [file Image3.tif]

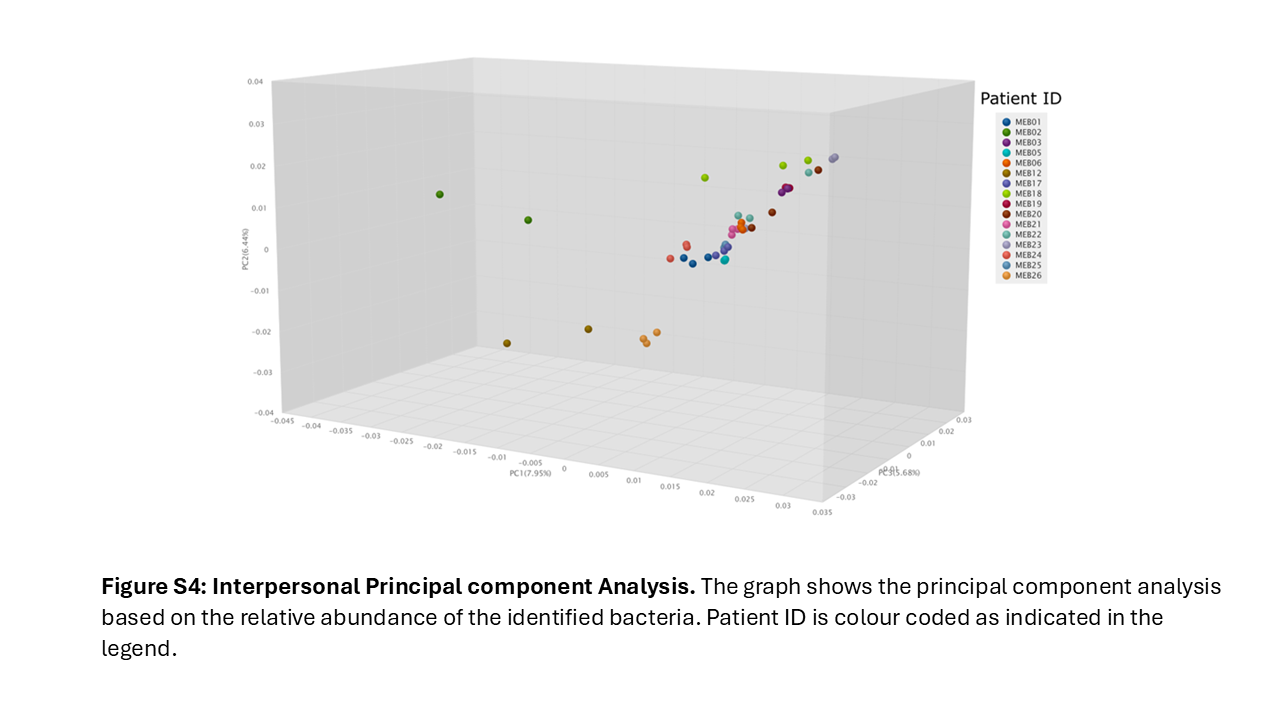

Supplement: Supplementary file 4 [file Image4.tif]

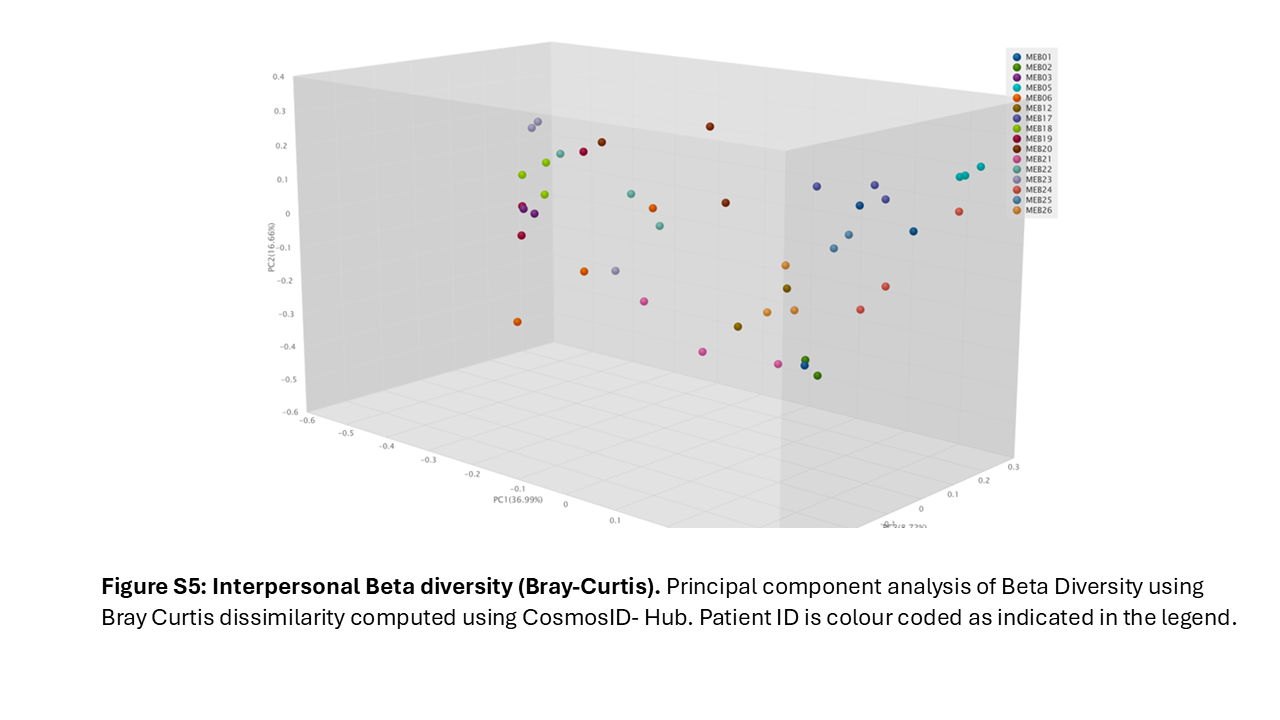

Supplement: Supplementary file 5 [file Image5.tif]

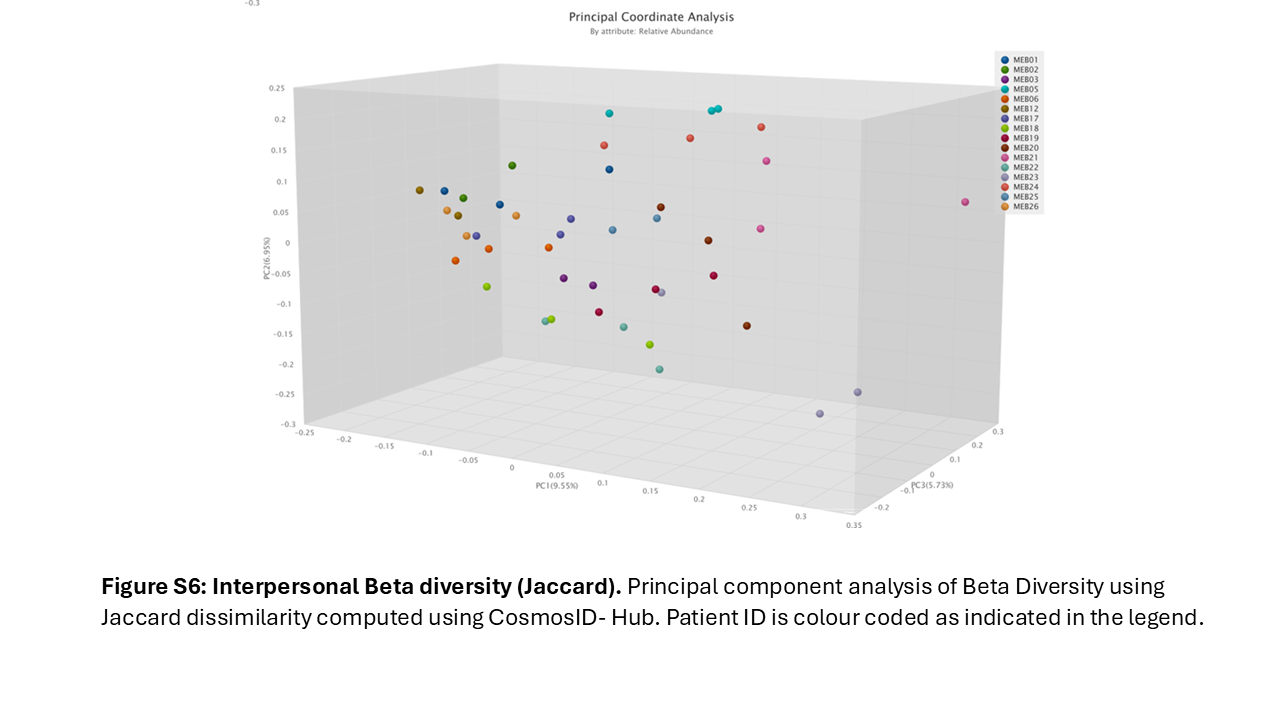

Supplement: Supplementary file 6 [file Image6.tif]

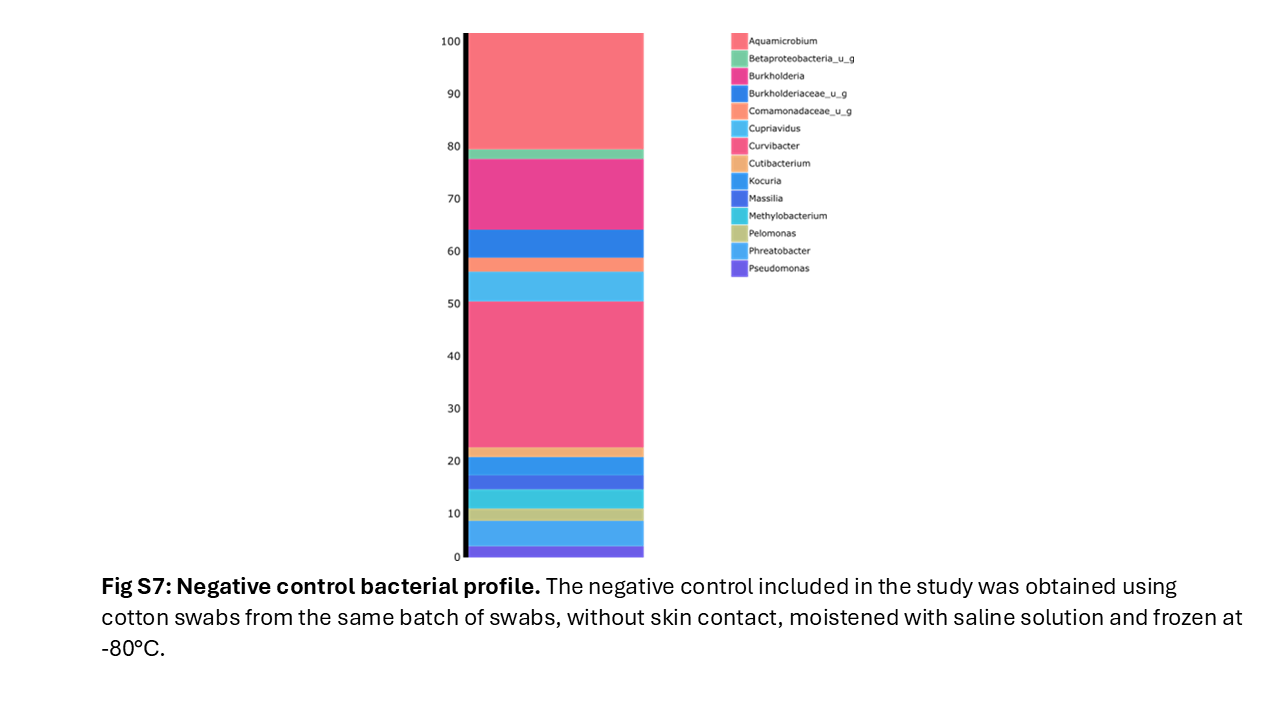

Supplement: Supplementary file 7 [file Image7.tif]
